# Construction of three-dimensional weather radar data from volcanic eruption clouds

**DOI:** 10.1016/j.mex.2021.101535

**Published:** 2021-09-30

**Authors:** Masayuki Maki, Takehiko Kobori

**Affiliations:** a Rsearch and Education Center for Natural Hazards, Kagoshima University. 1-21-40 Korimoto, Kaoshima 890-0065, Japan; b Sakurajima Volcano Research Center, DPRI, Kyoto University. 1722-19 Sakurajima-Yokoyama, Kagoshima 891-1419, Japan; c Graduate School of Science and Engineering, Kagoshima University. 1-21-40 Korimoto, Kagoshima 890-0065, Japan

**Keywords:** Advection vector, Interpolation, Sakurajima, Visualization, Pattern matching

## Abstract

Analysis tools of three-dimensional weather radar data (ANT3D) was originally developed at the National Research Institute for Earth Science and Disaster prevention (NIED) to retrieve three-dimensional (3D) precipitation and wind fields for convective storms. In 2013, Kagoshima University significantly revised ANT3D for analyses of volcanic eruption clouds, mainly to improve the temporal and spatial interpolation of radar data and estimation of the advection vector, which is required for temporal interpolation. Detailed information pertaining to these algorithms is listed as additional information in this paper.

• Procedures necessary for the construction of three-dimensional (3D) volcanic cloud weather radar data are described.
• An algorithm based on temporal and elevation angle interpolation methods was used to create 3D constant altitude plan position indicator (3D CAPPI) data with high temporal and spatial resolution.
• Two programs (ANT3D_GUI and the CAPPI viewer) are provided for readers interested in analyzing volcanic eruption cloud radar data.

## Graphical abstract

**Figure:**
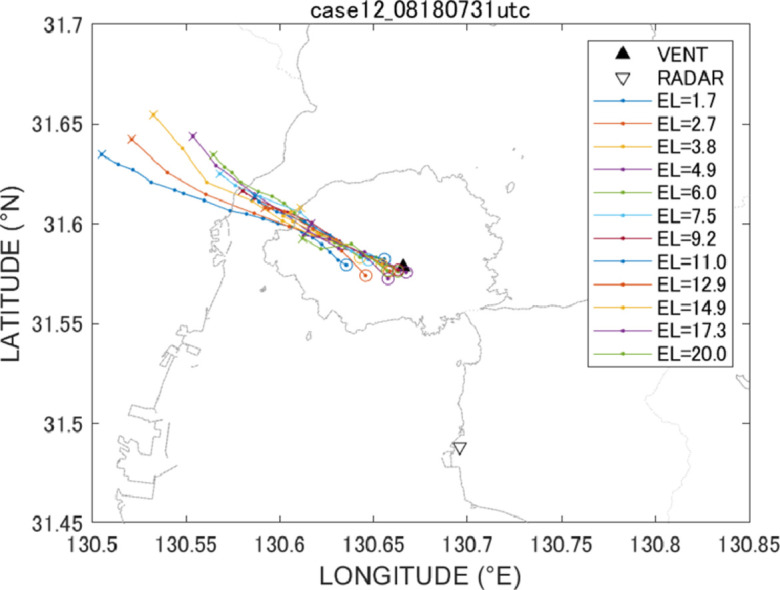

Specifications table

**Table:** 

Subject area:	Earth and planetary sciences
More specific subject area:	Volcanic eruption
Method name:	Analysis tools of three-dimensional weather radar data (ANT3D)
Name and reference of original method:	N.A.
Resource availability:	T. Kobori, “ant3d_gui”, Mendeley Data, v1 (2020), http://dx.doi.org/10.17632/7hmrbbbv39.1

**Method details**

## Background

Weather radar data are useful for monitoring volcanic eruption clouds [1], [2], [3], [4], Both research radars and operational weather radars, which are used for measuring rainfall, can be used for detection and ranging of volcanic ash falls. For example, the X-band polarimetric radar used in the present study, which is located approximately 11 km from the active Sakurajima volcano, has been collecting eruption cloud data since 2012. Although the radar can monitor volcanic eruption clouds spreading across a wide area and provides useful information regarding the eruption cloud structure, difficulties concerning radar measurements of such clouds include low temporal and spatial resolution, especially in the vertical direction, which are unsuitable for three-dimensional measurements of rapidly developing eruption clouds. The low temporal resolution arises because radar (except advanced research radar such as a phased-array radar) generally requires 5 to 10 min to acquire three-dimensional data regarding a volcanic eruption cloud. The low spatial resolution in the vertical direction occurs because the elevation angle interval is restricted by the predetermined period of volume scanning.

This paper presents algorithms, based on interpolation and image morphology, that overcome the aforementioned deficiencies of radar. It also presents some filtering techniques necessary for radar data quality control and construction procedures for three-dimensional CAPPI data.

## Outline of the analysis tools of three-dimensional weather radar data (ANT3D)

**Fig. 1:**
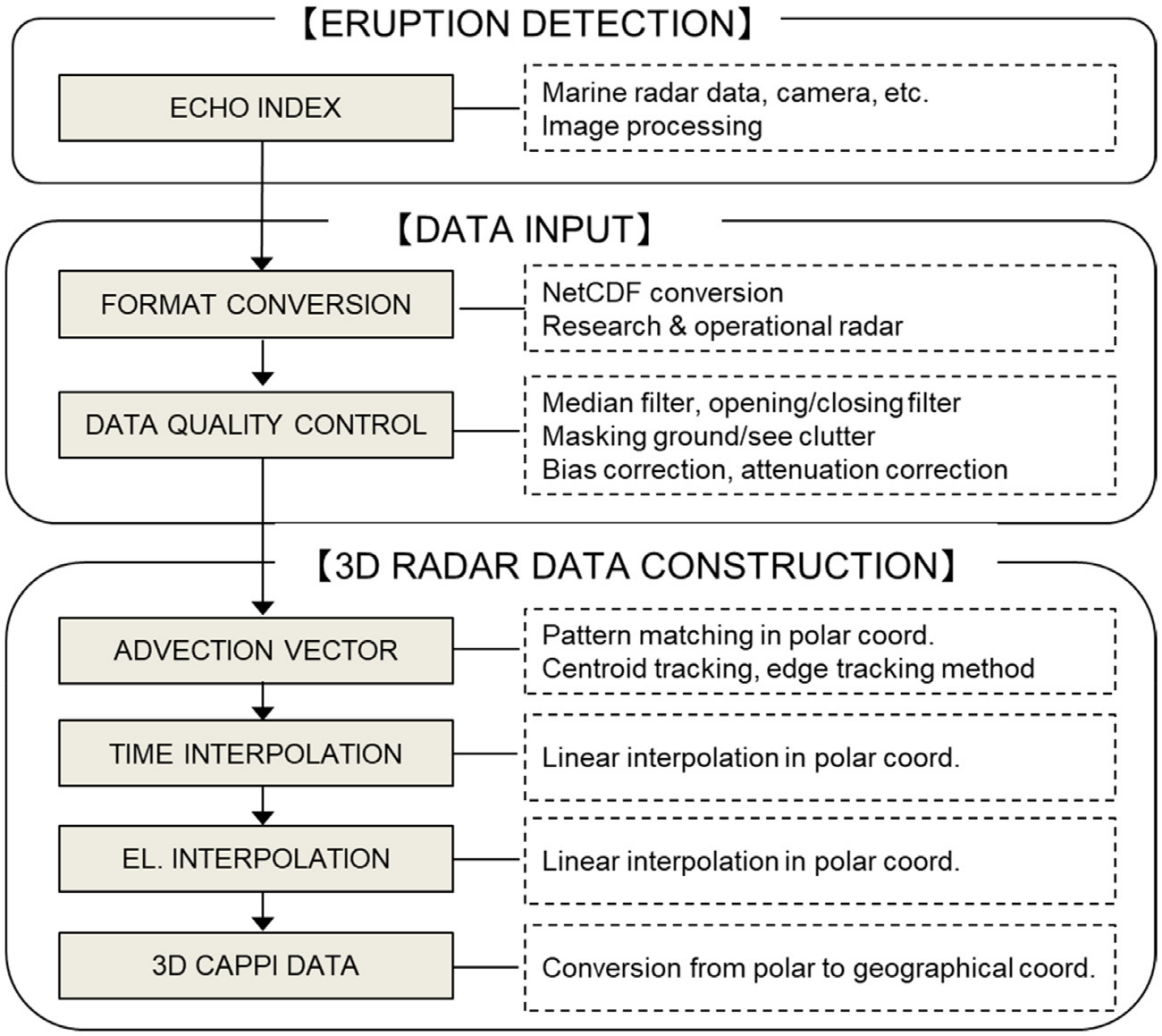
The flowchart of radar data analysis procedures used in the analysis tools for three-dimensional weather radar data (ANT3D) [3].

The main components of ANT3D are summarized in Fig. 1. The first component, eruption detection, is under development. In this component a marine radar, with fast vertical scanning and a fan beam antenna, will be used for the detection of eruptions over a vent and to estimate upward motion and eruption column height. When an eruption is detected, the weather radar data will be collected, converted to NetCDF data, and controlled for quality in the DATA INPUT component. The 3D CAPPI radar data are constructed in the 3D radar data construction component using a set of two-dimensional PPI data collected by weather radar. Calculations of 3D CAPPI data, which are necessary for 3D analyses and the visualizations of eruption columns, are basically done by interpolation and coordinate transformation schemes. The detailed of each component are presented in the following sections of the present paper.

## Quality control of radar data

### Noise filter

Table 1 lists the noise filters used in the present study. All listed filters are commonly used in image processing (e.g., [5]) and can be applied to the processing of radar data. The threshold filter is the simplest filter; it unsets data points that are outside (or inside) of a preassigned value. The threshold filter is also commonly used in binary image processing, as discussed below. Morphological processing removes small spots, holes, and dents in an object without changing the shape of the object. A typical example of morphological processing is a median filter, which substitutes a pixel value of focus with the median value of neighborhood pixel values. Other morphology operations include dilation and erosion. The dilation operation sets a value of “1” to a focus pixel if any neighborhood pixel has a value of 1. By applying this process to all pixels of an image, all holes in the image are filled. Note that a pixel is added at the image boundary because of the dilation operation (i.e., the image is expanded). In contrast, the erosion operation sets a value of “0” to a focus pixel if any neighborhood pixel has a value of 0. By applying this process to all pixels of an image, all spots in the image are removed. Because of the erosion operation, a pixel is removed at the image boundary (i.e., the image is contracted).

**Table 1:** List of typical noise filters.

Filter	Explanation	Values
Thresholding	Sets signals to zero if they are below a threshold value.	5 dBZ (default)
Median (or majority)	Removes objects that are excessively small, as well as holes, gaps, bays, and peninsulas; does not generally change object size or background.	3 × 3 (default)
Dilation	Fills pores and cracks in connected components of an image using an appropriate structuring element.	Once (default)
Erosion	Removes any noise-like pixel and small projection scattering in the analyzed image background.	Once (default)
Opening	Applies dilation following erosion to a binary image to remove noise-like 1-value pixels.	Once (default)
Closing	Applies erosion following dilation to a binary image to remove noise-like 0-value pixels.	Once (default)
Masking	Sets undesired portions of the binary image to 0-value pixels.	See Fig. 6

The dilation and erosion operations, which fill a hole and remove a spot, respectively, change the image shape. To restore the image shape, the image contracted by erosion operations is expanded by the same number of dilation operations; this is considered the opening operation. Conversely, the image expanded by dilation operations is contracted by the same number of erosion operations; this is considered the closing operation.

**Fig. 2:**
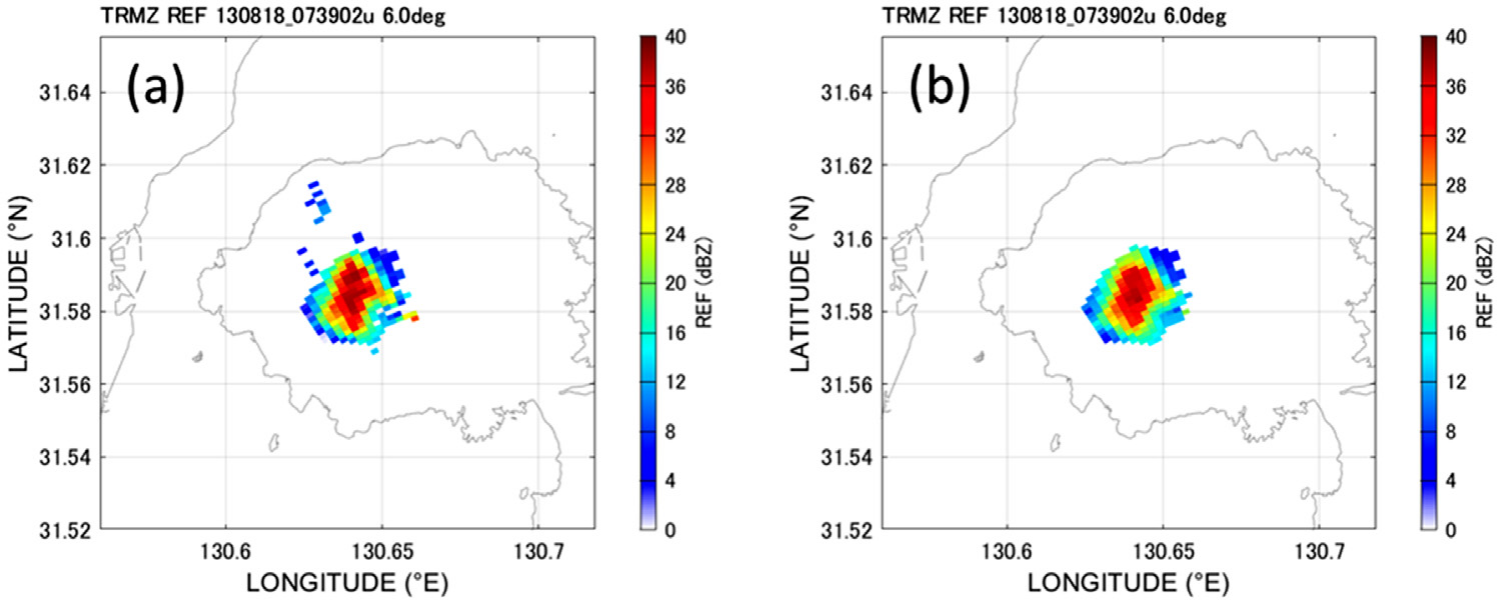
(a) Observed radar image and (b) radar image after noise filtering.

Fig. 2 demonstrates the effect of noise filtering on radar data. Holes in the target echo and spots outside of the echo are removed by the median filter, as well as opening and closing processing.

### Range side lobe filter

**Fig. 3:**
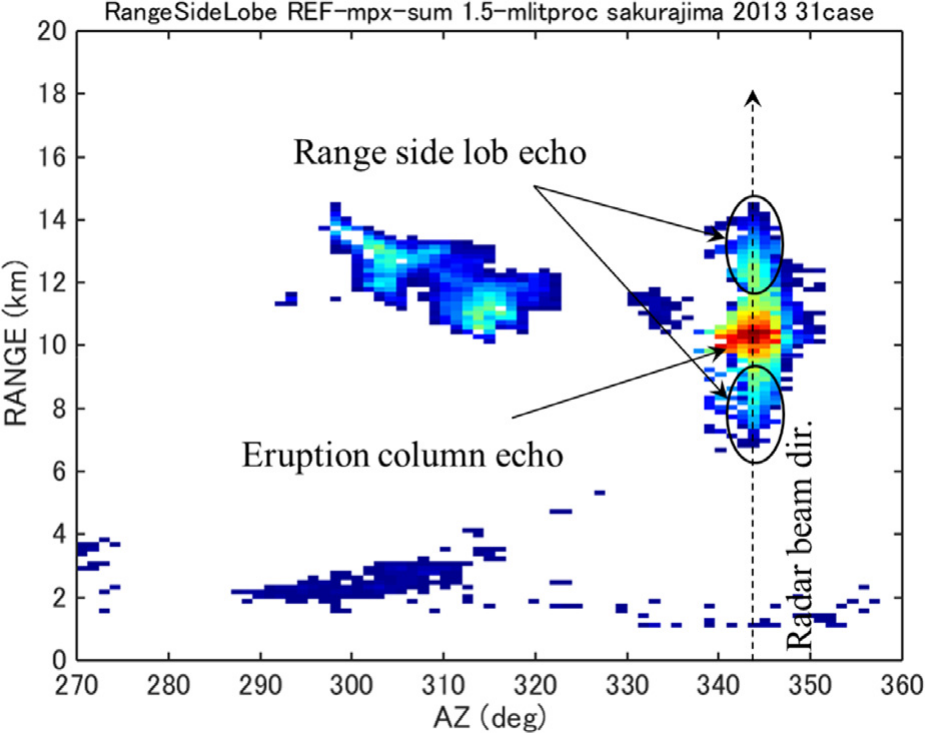
Example of range side lobe echoes of pulse compression radar (sum of reflectivity factors of 31 Sakurajima eruptions in 2013).

The range side lobe is a phenomenon that occurs with pulse-compressed radar. The range side lobe echo contaminates a true eruption echo and may lead to overestimation of the ash fall amount and area. Fig. 3 shows an example of a range side lobe echo. Range side lobe echoes were recognized in a total of 31 Sakurajima eruption cases in 2013, all of which exhibited an eruption column height greater than 3,000 m.

**Fig. 4:**
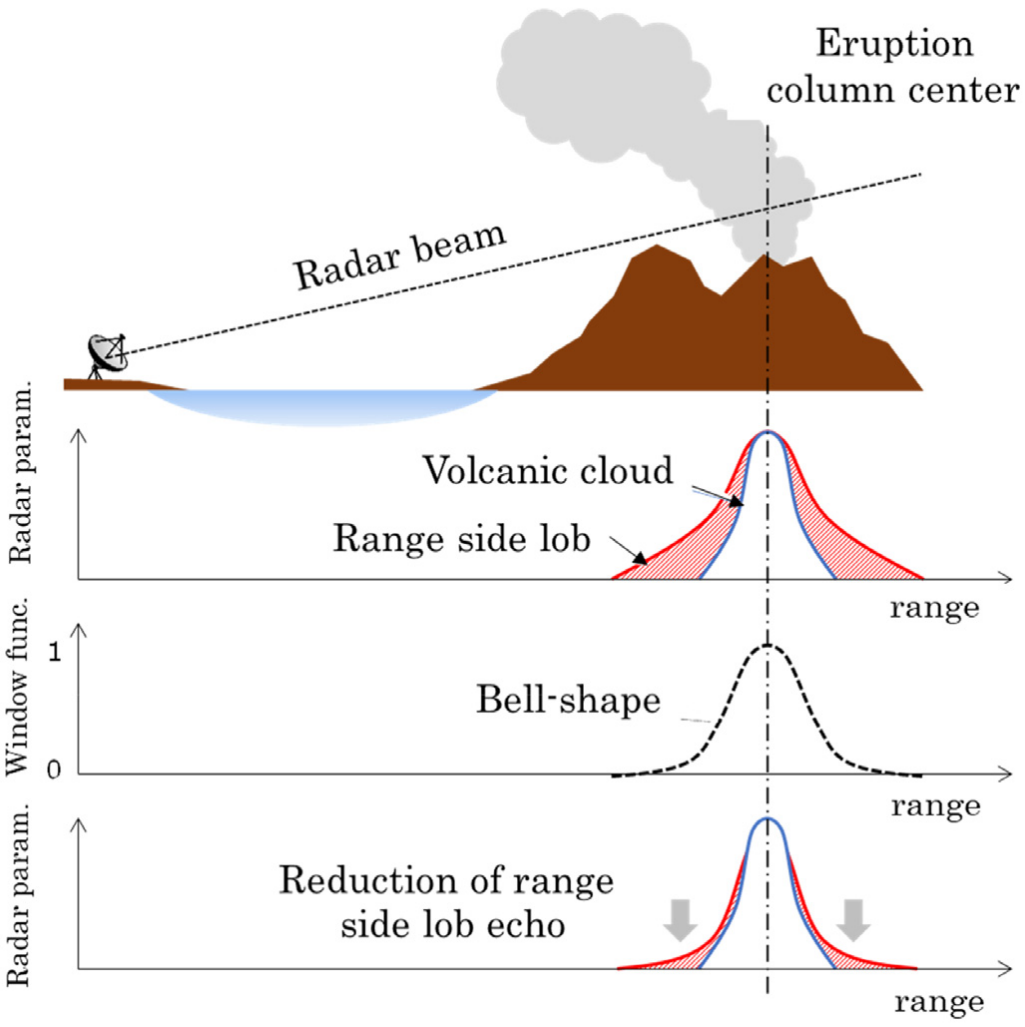
Schematic of range side lobe reduction filter.

**Fig. 5:**
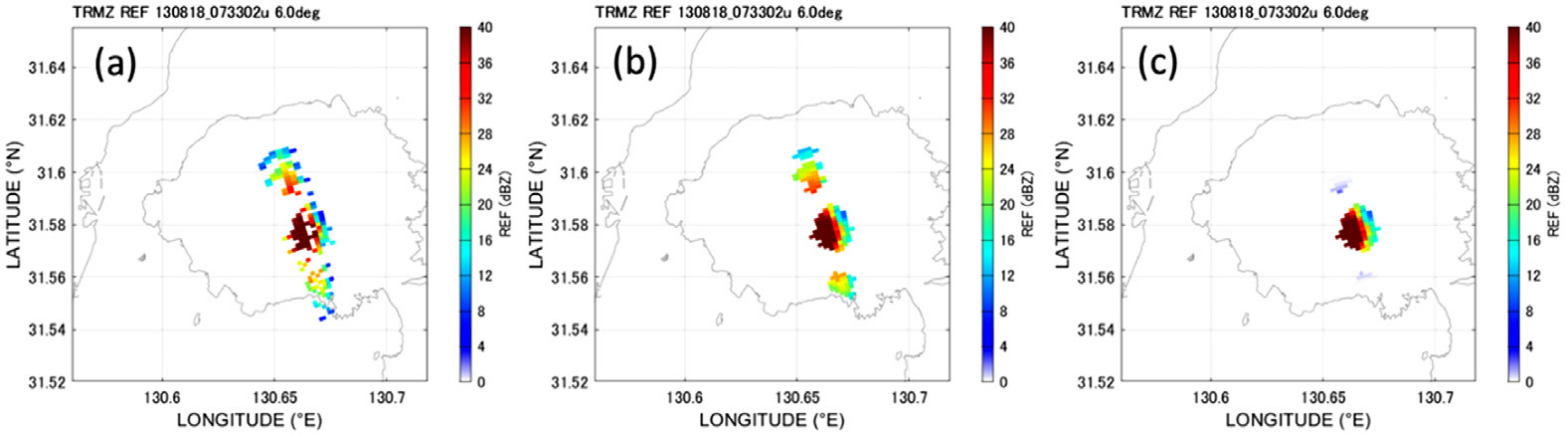
Radar echo of eruption column–(a) Observed, (b) after noise filtering, and (c) after range lobe echo filtering (bell-shaped window function).

The procedure for removing the range side lobe echo consists of noise filters, such as the median filter and opening and closing filters listed in Table 1, as well as the bell-shaped window function filter. Fig. 4 shows a schematic of the range side lobe echo, a bell-shaped window function, and the result of the filtering. Fig. 5 shows actual results of filtering. As shown in Fig. 5(a), the radar echo observed immediately after eruption includes a spurious echo radiating from the strong echo of the eruption column over the vent. The echo also contains holes and spots that should be removed. After the median filter is applied to the observed image, the holes and spots are filled and removed, respectively, and the image is smoothed (Fig. 5(b)). However, the range side lobe echo remains. The range side lobe echo is effectively removed by the bell-shaped window function, as shown in Fig. 5(c). The bell-shaped window is fixed over the vent. Thus, range side lobes other than from the vent are not removed. The parameters of the bell-shaped window were determined statistically from the range side lobs observed in a total of 31 eruptions in 2013: The position, range width, and azimuthal width of the bell-shaped window function are 10.5 km, 2 km, and 10°, respectively.

### Masking

**Fig. 6:**
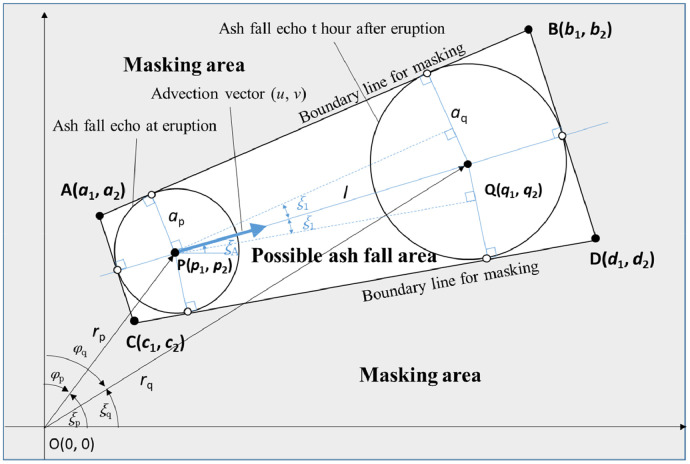
Schematic of a volcanic eruption cloud trajectory, its ash fall area, and its masking area. The earth's curvature is ignored. O(0,0)–Position of radar, P(p_1_,p_2_)–Center of volcanic eruption cloud at time of eruption, Q(q_1_,q_2_)–Center of volcanic eruption cloud at time *t* after eruption, *a*_p_–Radius of volcanic eruption cloud at P, *a*_q_–Radius of volcanic eruption cloud at Q, (u,v)–Components of advection vector, *ξ*_A_ direction of advection vector (mathematical angle), *l*–Distance between P and Q, (*r*_p_,*ξ*_p_)–Polar coordinates of P (mathematical angle), (*r*_q_,*ξ*_q_)–Polar coordinates of Q (mathematical angle), (*r*_p_,ϕ_p_)–Radar coordinates of P, (*r*_q_,*ϕ*_q_)–Radar coordinates of Q.Shadowed area is masking area.

Ground clutters and sea clutters, which contaminate eruption cloud echoes, constitute an obstacle to analyses of target echoes because they clutter and obscure the echo. Masking is a simple method for reducing the effects of clutter echoes on target echoes. A typical clutter filter comprises the moving target indicator, which is built into the signal processing unit of a radar system. An alternative clutter filtering method involves the use of a clutter map based on a topographical map or actual clutter echoes observed by radar. With respect to volcanic eruption cloud observations by radar, precipitation also produces an echo that is undesirable for volcanic eruption cloud analyses. In this study, automatic masking was implemented, based on the possible volcanic eruption cloud trajectory. Fig. 6 shows a schematic of the trajectory: a volcanic eruption cloud, its ash fall area, and its masking area. Notably, the non-masking area (within trapezoid A, B, C, D) can be determined by geometrical calculations if the advection vector (*u,v*) and radii *a*_p_ and *a*_q_ of the volcanic ash cloud are known or assumed.

## Algorithms of advection vector of volcanic clouds

### Outline

After an eruption, volcanic eruption clouds move and diffuse downwind, depending on the local wind direction. As the local wind changes vertically, the three-dimensional shape and structure of the cloud change vertically. This section shows that the vertical wind profile can be estimated from advection vectors of a volcanic eruption cloud on PPI at different elevation angles. Studies on the estimation of advection vector of precipitation echoes can be classified into two categories: Lagrangian method [5], [6], [7], [8], [9], [10] and Eulerian method [11], [12], [13], [14]. The centroid tracking method belongs to the Lagrangian category and the pattern matching method belongs to the Eulerian category.

In the following sections, we describe two kinds of advection vector algorithms: centroid chasing and pattern matching methods. Then, we discuss their possible applications for retrieval of the vertical wind profile and the volcanic ash fall nowcast. Application of the advection vector to the construction of three-dimensional CAPPI data is explained in detail in the next chapter.

### Centroid tracking method

The centroid position $\left(r_{g},\;\phi_{g}\right)$ at a PPI plain with the *k*-th elevation angle $\theta_{k}$ of a volcanic eruption cloud can be calculated from the following equations:

(1) $$\begin{array}{ccc} r_{g} & = & \sum_{\phi =0}^{n-1}\sum_{r=0}^{m-1}r\times f\left(r,\phi ,\theta_{k}\right)/\sum_{\phi =0}^{n-1}\sum_{r=0}^{m-1}f(r,\phi ,\theta_{k})\\ \phi_{g} & = & \sum_{\phi =0}^{n-1}\sum_{r=0}^{m-1}\phi \times f\left(r,\phi ,\theta_{k}\right)/\sum_{\phi =0}^{n-1}\sum_{r=0}^{m-1}f(r,\phi ,\theta_{k}) \end{array}$$

where $f(r,\phi ,\theta_{k})$ is the radar reflectivity factor of the PPI radar echo; r, ϕ, and $\theta_{k}$ are the range, azimuth angle, and elevation angle of the *k*th PPI scan, respectively; and n and m are the total numbers of data points in the range and azimuth directions, respectively.

**Fig. 7:**
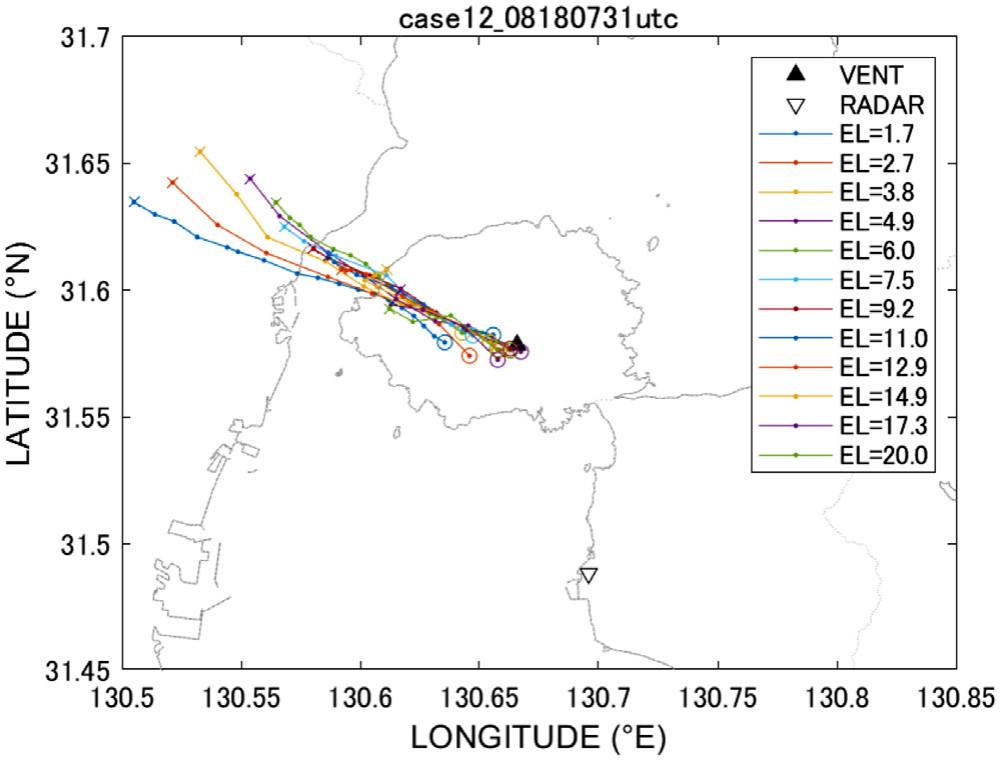
Centroid trajectories at PPI elevation angles of 1.7°, 2.7°, 3.8°, 4.9°, 6.0°, 7.5°, 9.2°, 11.0°, 12.9°, 14.9°, 17.3°, and 20° for the Aug. 18, 2013 eruption.

The advection vector components $\left(v_{r},v_{\phi }\right)$ on the azimuth–range coordinate system are obtained by chasing centroid positions of the target echoes and can be expressed as follows:

(2) $$\begin{array}{ccc} v_{r} & = & \frac{\partial r}{\partial t}=\frac{r_{i}-r_{r-1}}{t_{i}-t_{i-1}}\\ v_{\phi } & = & \frac{\partial \phi }{\partial t}=\frac{\phi_{i}-\phi_{{}_{i-1}}}{t_{i}-t_{i-1}} \end{array}$$

where $\left(r_{i},\phi_{i}\right)$ are the advection components on the azimuth–range coordinate system at time $t_{i}$. Note that *i* = 0 and *i* = *n*_i_ indicate the times of eruption and when radar echoes disappear. By applying the above procedures to all elevation data (k = 1, *n*_k_), we can obtain vertical profiles of $\left(v_{r},v_{\phi }\right)$. Fig. 7 shows an example of the analyses, in which centroid trajectories were obtained for different PPI data concerning the August 18, 2013, Sakurajima eruption. If we can obtain some information from the movements of the radar echoes we can use such information to improve the accuracy of ash fall transportation models. We will show in the later section of the present paper how to retrieve a vertical profile of local winds using the advection vector,

### Pattern matching method

The pattern matching method, also known as the correlation method, estimates the advection vector by identifying the position that minimizes the normalized cross-correlation between two sequential PPI images of volcanic eruption clouds. The normalized cross-correlation coefficient $C_{fg}$ is defined as follows:

(3) $$C_{fg}\left(r,\phi ,\gamma ,\eta \right)=\frac{\sum_{0}^{n-1}\sum_{0}^{m-1}\left\{f\left(r+i,\phi +j\right)-\bar{f}\right\}\left\{g\left(r+i+\gamma ,\phi +j+\eta \right)-\bar{g}\right\}}{\sqrt{{\sum_{0}^{n-1}\sum_{0}^{m-1}\left\{f\left(r+i,\phi +j\right)-\bar{f}\right\}}^{2}}\sqrt{{\sum_{0}^{n-1}\sum_{0}^{m-1}\left\{g\left(r+i+\gamma ,\phi +j+\eta \right)-\bar{g}\right\}}^{2}}}$$

where $\bar{f},\;\bar{g}$ are the areal sum of reflectivity factors f(r,ϕ) and g(r,ϕ) at times t and *t* + Δ*t*, respectively, and are defined by the following equations:

(4) $$\begin{array}{ccc} \bar{f} & = & \frac{\sum_{0}^{n-1}\sum_{0}^{m-1}f(r+i,\phi +j)}{nm}\\ \bar{g} & = & \frac{\sum_{0}^{n-1}\sum_{0}^{m-1}g(r+i+\xi ,\phi +j+\eta )}{nm} \end{array}$$

The parameters *γ* and *η* are control parameters of the range *r* and azimuth angle *ϕ*, respectively. In the method, $C_{fg}$ is calculated by changing the values of *γ* and *η* to find the matching points. The values *γ* and *η* resulting in the maximum $C_{fg}$ provide these matching points. The components of the advection vector are given by the following equations:

(5) $$\begin{array}{c} v_{r}=\gamma /\Delta t\\ v_{\phi }=\eta /\Delta t \end{array}$$

**Fig. 8:**
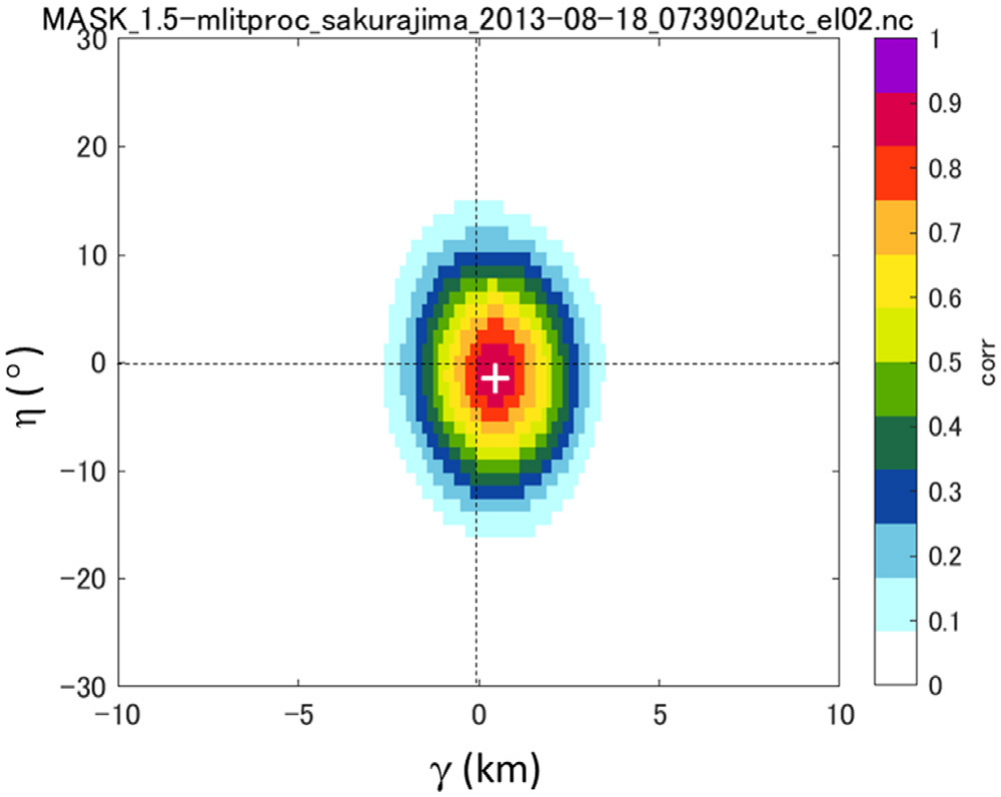
Distribution of $C_{fg}$ on the *γ* –*η* coordinate system (16:39:02 LST, August 18, 2013, Sakurajima eruption). The PPI elevation angle is 6.0°.

Fig. 8 shows an example distribution of $C_{fg}$ on the *γ*–*η* coordinate system. The white ‘+’ symbol shows the position of the maximum $C_{fg}$, i.e., the best matching distance of the range and azimuth angle of two images. By repeating the calculations above for all elevation angles $\left(\theta_{k},\;k=1,n_{k}\right)$, advection vectors are obtained for each elevation angle.

### Functional form of advection vector

The advection vector can be obtained at every observation time *t* and every elevation angle *θ* (i.e., the advection vector can be expressed by a function of time *t* and *θ*). In this section, we obtain its adequate functional form. We assume that the advection vector is expressed by the following linear equations of *t*:

(6) $$\begin{array}{c} v_{r}\left(\theta ,t\right)=m_{r}\left(\theta \right)\;t+n_{r}\left(\theta \right)\\ v_{\phi }\left(\theta ,t\right)=m_{\phi }\left(\theta \right)\;t+n_{\phi }\left(\theta \right) \end{array}$$

where $v_{r}$ and $v_{\phi }$ are the range (*r*) and azimuth (*ϕ*) components of the advection vector, respectively. The parameters $m_{r}$ and $n_{r}$ are the coefficient and constant of $v_{r}$, respectively; they can be obtained by regression analyses. Similarly, the parameters $m_{\phi }$ and $n_{\phi }$ are the coefficient and constant of $v_{\phi }$, respectively; they can also be obtained by regression analyses. Next, we assume that $m_{r}$, $n_{r}$, $m_{\phi }$, and $n_{\phi }$ are expressed by the following polynomial expressions of *θ* to find a functional form:

(7) $$\begin{array}{ccc} m_{r}\left(\theta \right) & = & \sum_{i=1}^{n}a_{r_{i}}\theta^{i-1},\;\;n_{r}\left(\theta \right)=\sum_{i=1}^{n}b_{r_{i}}\theta^{i-1}\\ m_{\phi }\left(\theta \right) & = & \sum_{i=1}^{n}c_{\phi_{i}}\theta^{i-1},\;\;n_{\phi }\left(\theta \right)=\sum_{i=1}^{n}d_{\phi_{i}}\theta^{i-1} \end{array}$$

where $a_{r_{i}}$, $b_{r_{i}}$, $c_{\phi_{i}}$, and $d_{\phi_{i}}$ are coefficients obtained by regression analyses of $m_{r}$, $n_{r}$, $m_{\phi }$, and $n_{\phi }$, respectively.

**Fig. 9:**
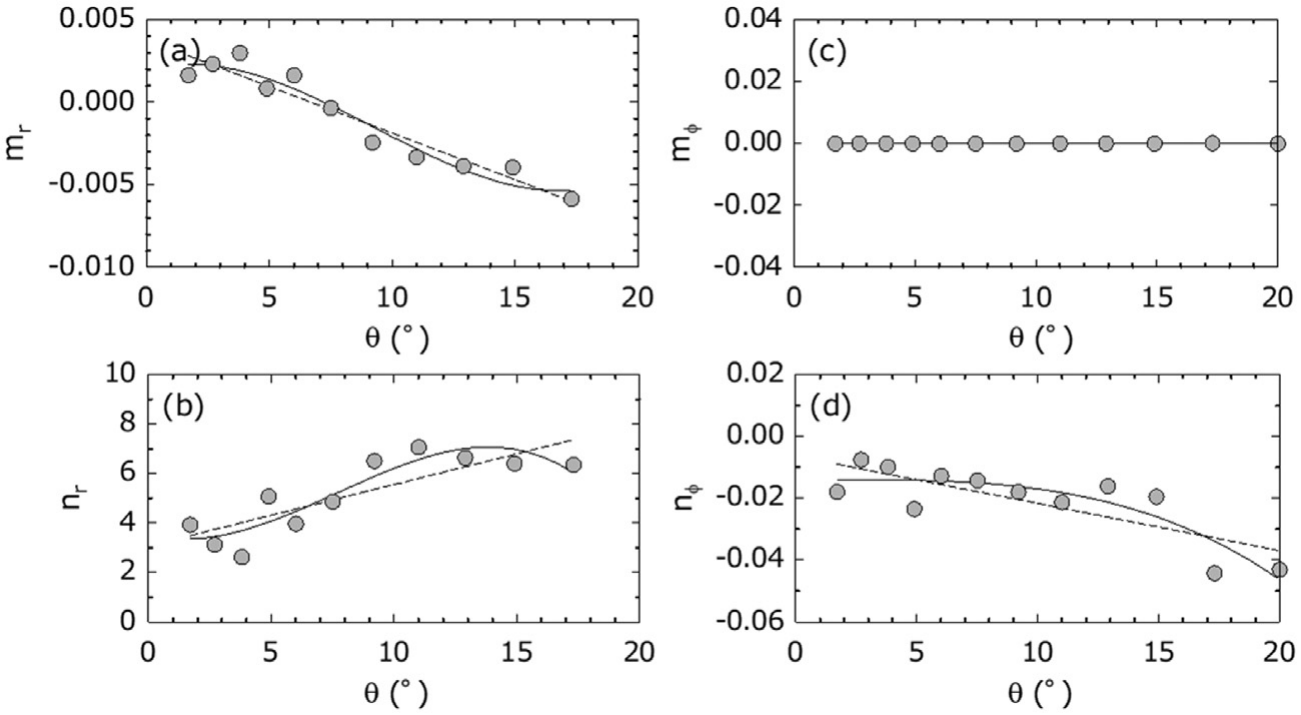
Observed $m_{r}$, $n_{r}$, $m_{\phi }$, and $n_{\phi }$ and their regression lines (solid line is for third polynomial and broken straight line is for linear relationship). Data: August 18, 2013, Sakurajima eruption.

Fig. 9 shows an example of the results of analyses: observed values of $m_{r}$, $n_{r}$, $m_{\phi }$, and $n_{\phi }$ are shown along with their regression curves. Comparisons of the observed values with the regression curves show that the third-order polynomial line (*n* = 3) is a better fit for the observed values, compared with the linear line (*n* = 1). Although the fifth-order polynomial line better explains the change of observed values (data not shown), the third-order polynomial line is a better practical choice because the available sample number for regression analyses depends on the lifetime of volcanic eruption clouds. When the lifetime is shorter, the sample number is smaller. If the local wind is simple, small sample numbers may be acceptable for regression analyses. It should be noted that the advection parameters were arbitrarily calculated for every two sequential images, then regression analysis was applied to their temporal change and elevation angle changes.

**Fig. 10:**
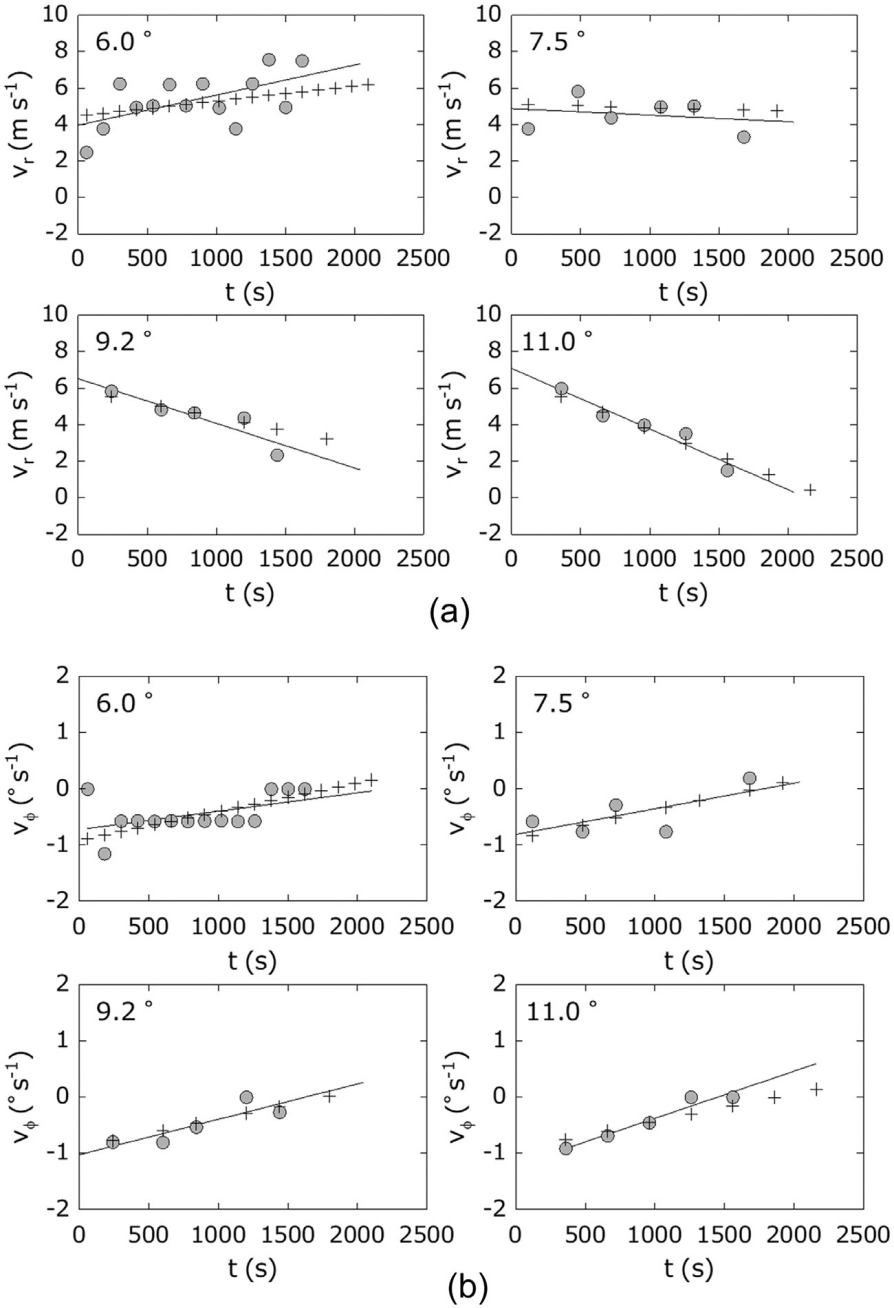
(a) Temporal change of range component $v_{r}$ of advection vector: observed value, +: calculation by (5). Straight line is regression line for observed values. Data–Aug. 18, 2013, Sakurajima eruption. (b) Similar to Fig. 9(a), except for altered $v_{\phi }$.

Fig. 10 shows temporal changes of the components of the advection vector ($v_{r}$, $v_{\phi }$) at elevation angles of 6.0°, 7.5°, 9.2°, and 11.0°, respectively. The estimated values determined by regression analyses agree with the observed values. Similar conclusions were obtained for other elevation angles.

### Improvements of advection vector

**Fig. 11:**
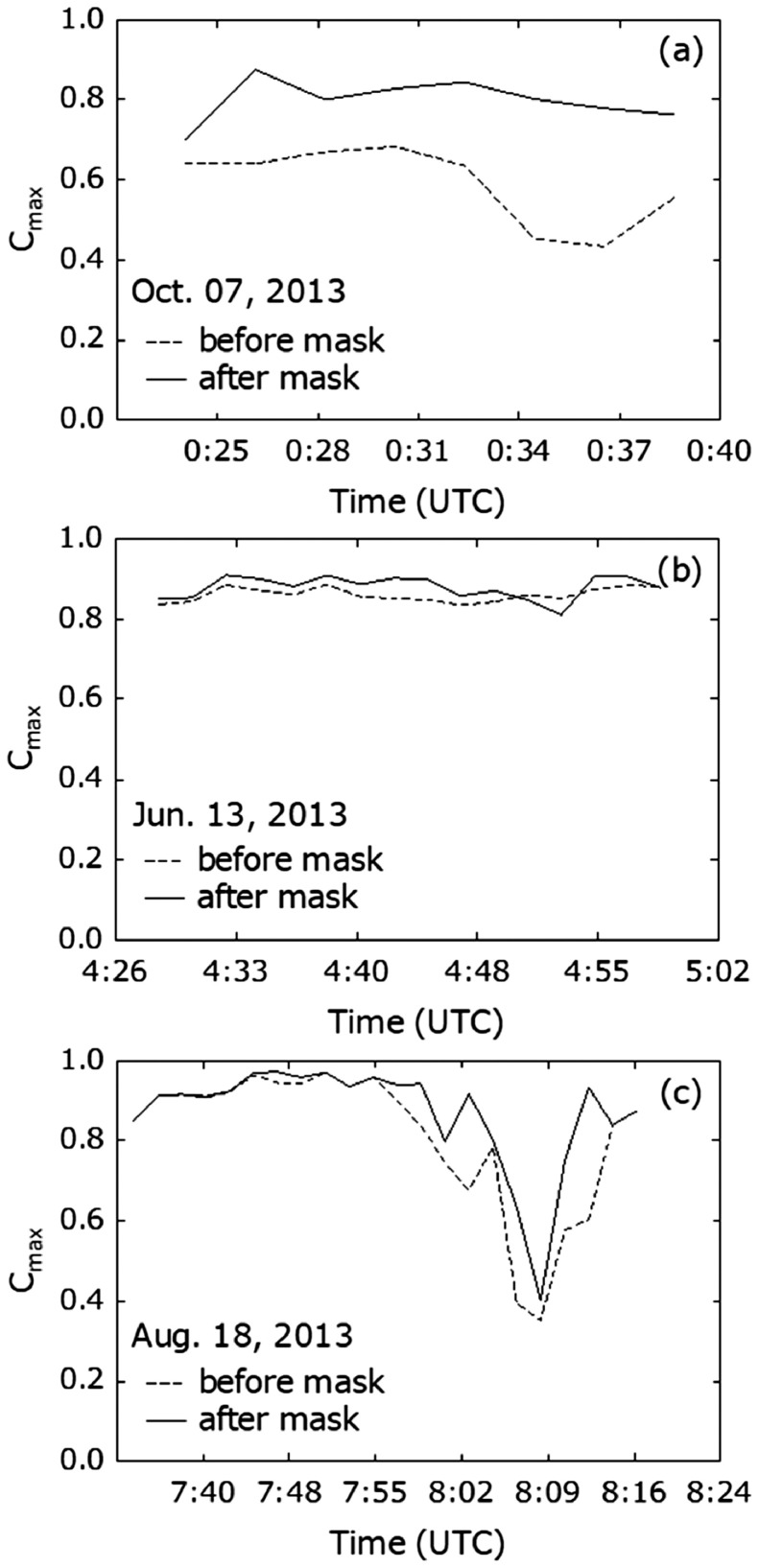
Temporal changes of C_max_ for Sakurajima eruptions on (a) Oct. 07, 2013, (b) Jun. 13, 2013, and (c) Aug. 18, 2013. Solid and dashed lines depict results with and without automatic masking shown in Fig. 5, respectively.

Both centroid chasing and pattern matching methods used in the advection vector estimations assume that a volcanic eruption cloud echo has a simple shape (e.g., an isolated shape). However, actual echoes are not always simple. Echoes from ground clutter, ships, and precipitation may coexist with or contaminate volcanic echoes. These echoes diminish the accuracies of estimated advection vectors. The noise filters, range side lobe filter, and masking mentioned in section 2 are essential for accurate estimation of advection vectors. Fig. 11 demonstrates improvement of the advection vector. The temporal change of C_max_ is used as the index of accuracy for the pattern matching method.

### Retrieval of vertical profiles of local winds

**Fig. 12:**
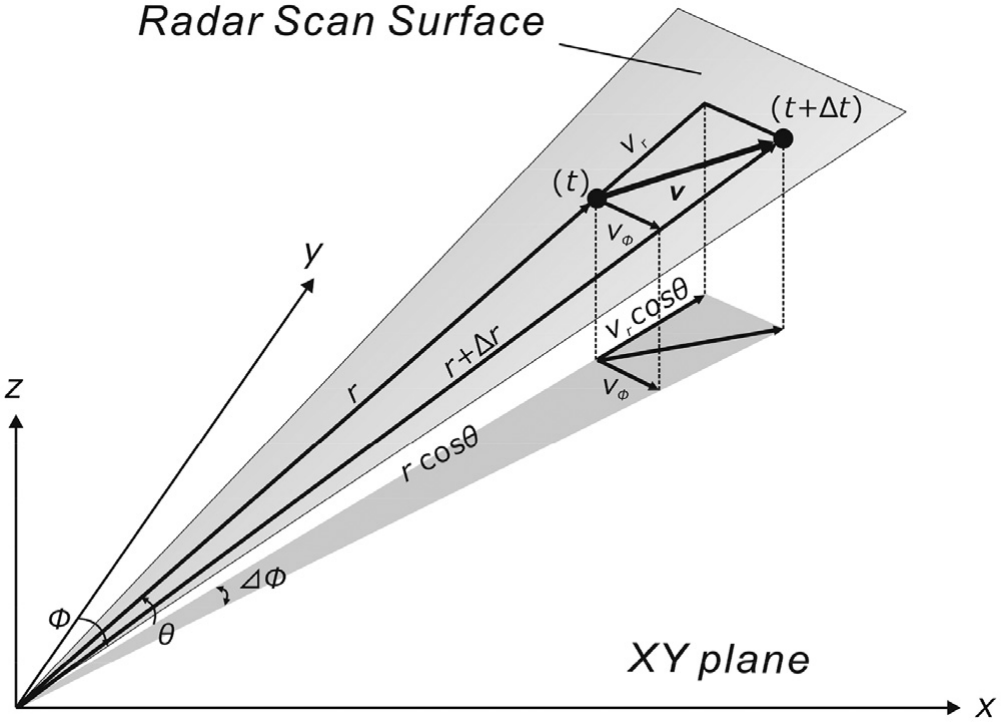
Calculation of wind components at elevation angle *θ*.

A useful application of the advection vector comprises retrieval of the vertical profile of local winds. If we can obtain some information on local winds from the movements of the radar echoes we can use such information to improve the accuracy of ash fall transportation models. Transforming the advection vector from the slant range–azimuth coordinate system (radar scan surface) to the plane rectangular coordinate system (XY plane), local wind components can be obtained by the following equations (see also Fig. 12):

(8) $$\begin{array}{c} v_{x}=v_{r}\cos\theta \sin\phi +r\cos\theta \;v_{\phi }\cos\phi \\ v_{y}=v_{r}\cos\theta \cos\phi -r\cos\theta \;v_{\phi }\sin\phi \end{array}$$

Because cosθ≈1 with respect to low-elevation PPI scans, (8) becomes

(9) $$\begin{array}{c} v_{x}=v_{r}\sin\phi +r\;v_{\phi }\cos\phi \\ v_{y}=v_{r}\cos\phi -r\;v_{\phi }\sin\phi \end{array}$$

By repeating the same procedure for all elevation angles, a vertical profile of wind speed and direction is obtained. Notably, the vertical wind component cannot be obtained.

### Identification of vertical axis of volcanic eruption column

Identification of the vertical axis of the eruption column is a challenge of using meteorological radar for monitoring of volcanic eruption clouds. When the eruption column height is greater than 10 km, the vertical motion caused by the eruption is dominant, compared to the horizontal motion caused by the local wind [15]. When the eruption column height is smaller than 5 km, the effect of local wind is dominant. When a vertical wind shear exists, the eruption column shape is distorted accordingly. When the eruption column height is greater than 10 km, the algorithm for detection of the eruption column centroid may be the key algorithm for identifying the vertical axis of the eruption column. When the eruption column height is smaller than 5 km, the retrieval algorithm of vertical wind profile is also a key algorithm for identifying the vertical axis. A vertical axis distorted by vertical wind shear may be visualized by connecting centroid points estimated at each height.

## Construction of three-dimensional CAPPI data

### Outline

Three-dimensional constant plan position indicator (3D CAPPI) data obtained from weather radar are useful for investigating the inner structure of volcanic eruption clouds. However, because of the mechanical scanning of a parabolic antenna, the temporal and spatial resolutions of radar data are insufficient for three-dimensional analyses of volcanic eruption clouds. A possible solution involves use of a research radar, such as a fast-scanning radar or a phased-array antenna radar. The other solution involves temporal and spatial interpolation of coarse sampling radar data. The key parameter for construction of three-dimensional CAPPI data is the advection vector of the volcanic eruption cloud. The methodological details are described in the following sections.

### Observed radar data

**Fig. 13:**
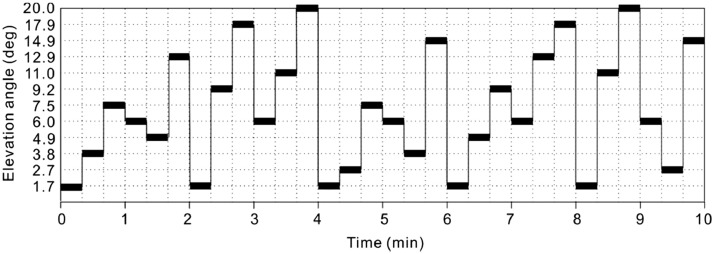
Example of a time table of PPI scans for three-dimensional observations of a volcanic eruption cloud. A black thick bar shows the scan period at each elevation angle.

Various antenna scanning modes are used in radars, depending on their tasks. Although research radar has a flexible scanning strategy (e.g., sectoral RHI scanning to reduce three-dimensional observation time), most operational radars have a fixed antenna scanning mode depending on the purpose of the radar. An example of a scanning strategy with the X-band polarimetric radar that was used in Maki et al. (2021) is shown in Fig. 13. A volume scan consisted of 12 tilt PPI scans. The scanning elevation angle is unnecessary in both ascending and descending orders. The MLIT designed an antenna PPI scan strategy that is used to obtain the precipitation distribution in the lower atmosphere, with higher temporal resolution. As shown in Fig. 13, PPI scans at the elevation angles of 1.7° and 6.0° are repeated at 2-min intervals, whereas other PPI scans are repeated at approximately 5 min intervals.

### Temporal and elevation interpolation

**Fig. 14:**
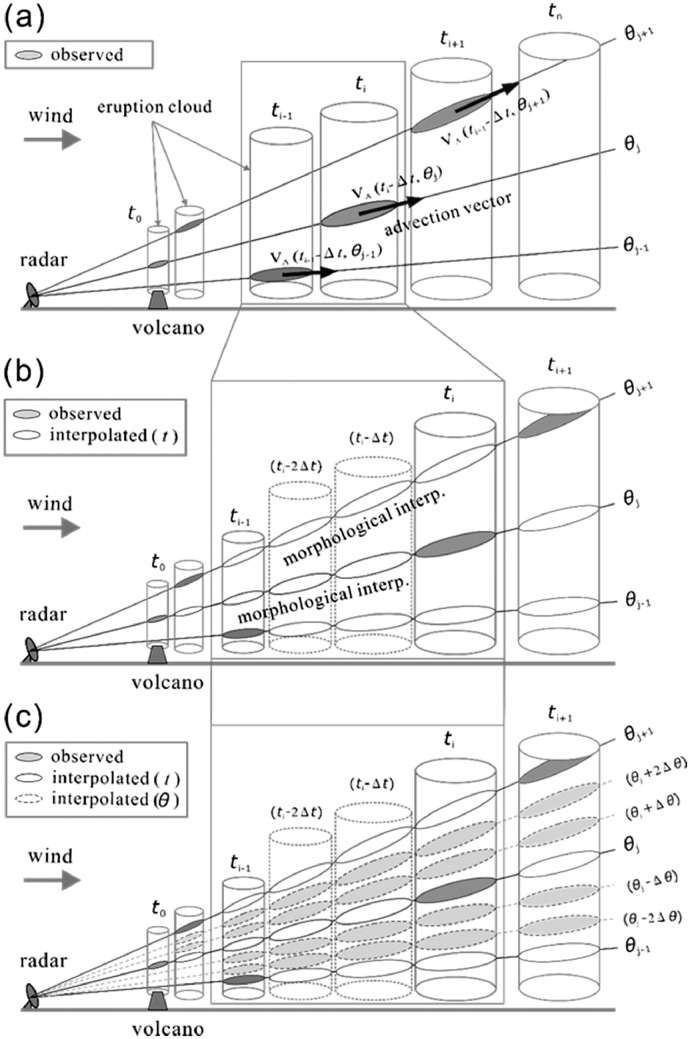
Schematic of radar data interpolation for the construction of three-dimensional CAPPI data of a volcanic ash cloud. (a) Observed echoes and their advection vectors, (b) morphological interpolation in the temporal domain, and (c) morphological interpolation in the azimuth angle domain. (after Maki et al., 2021 [3])

Fig. 14 (a) schematically shows volcanic eruption clouds moving in the local wind direction and beams of PPI at each elevation angle. The echoes observed by radar are shown by shaded ellipses. Notably, the radar cannot obtain all PPI images of a volcanic eruption cloud at a single time point because approximately 15 s are required to finish one PPI scan. Thus, as shown in Fig. 14(b), temporal interpolation is necessary to fill the data between two different observation times of a PPI scan. Advection vectors estimated by the methods described in the previous section are used for the temporal interpolation of the echo position. In addition, morphological interpolation is required to consider the change in interpolated echo shape. The morphological interpolation used in the present study is explained as follows. Using the estimated advection vector, The PPI image of *Z* observed at *t*_2_ can be positioned back to the place where it was observed at $t_{1}$. We can obtain ΔZ during *t*_2_-*t*_1_ at any point (*x*_i_, *y*_j_) by comparing images of Z observed at *t*_1_ and *t*_2_, respectively. Assuming linear temporal interpolation, we obtain the following formula to estimate the value at $t_{1}+\Delta t$.

$$Z_{t_{1}+\Delta t}\left(x_{i},y_{j}\right)+\left(Z_{t_{2}}\left(x_{i},y_{j}\right)-Z_{t_{1}}\left(x_{i},y_{j}\right)\right)\cdot \Delta t/\left(t_{2}-t_{1}\right)$$

After repeating the above procedures at all grid points, we can obtain a series of interpolated Z, (i.e., $Z_{t1+Dt},Z_{t1+2Dt},\ldots$), following which each estimated Z is placed at the appropriate positions calculated by the advection vector. It should be noted that the temporal interpolation is done in the slant surface. Importantly, interpolation is not possible unless two observed echoes are provided (e.g., during a few minutes following the eruption).

**Fig. 15:**
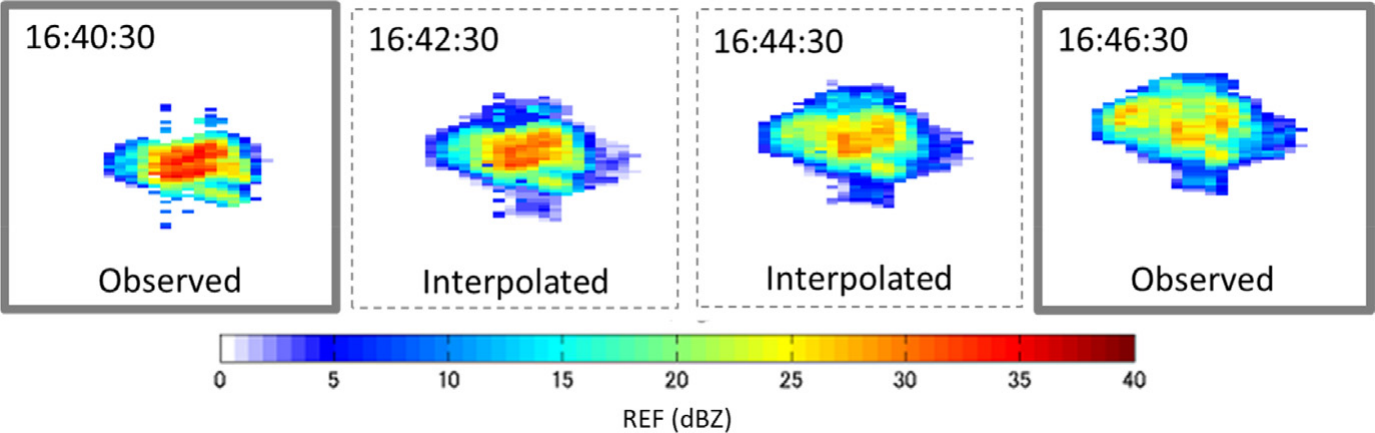
Interpolated PPI images at 30 s interval of time. August 18, 2013, Sakurajima eruption.

An example of temporal interpolation of radar echoes is shown in Fig. 15, where linear interpolation at 30 s intervals was applied to PPI echoes observed at the elevation angle of 7.5° for the Sakurajima eruption on August 18, 2013. As shown, the interpolated echoes smoothly connect the observed echoes.

**Fig. 16:**
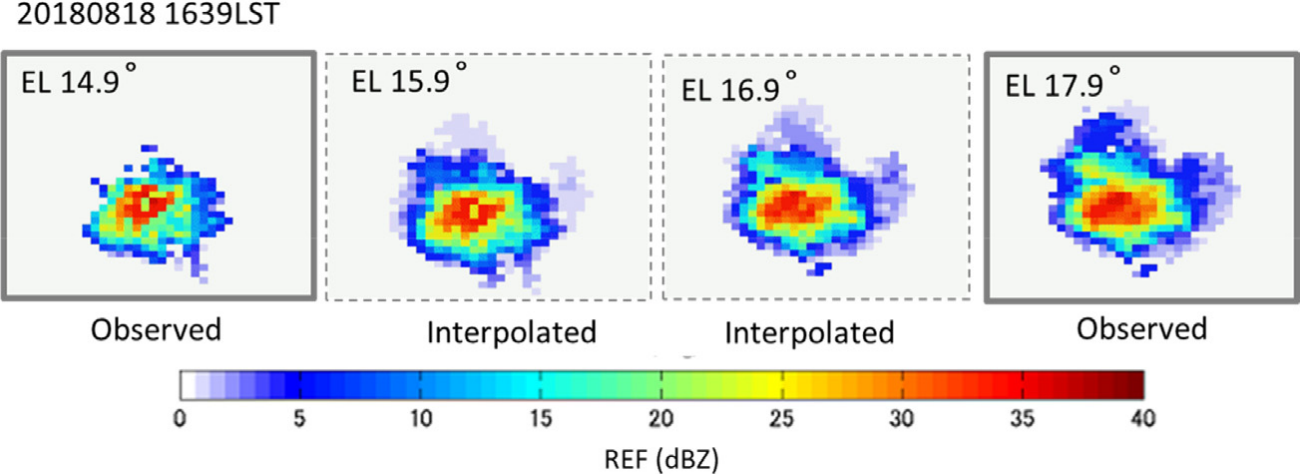
Interpolated PPI images at 30″ interval of elevation angle. 16:39 LST, August 18, 2013, Sakurajima eruption.

Because the temporally interpolated radar data remain coarse in the vertical direction, elevation angle interpolation is also necessary to represent the volcanic ash cloud structure. Using observed echoes and temporally interpolated echoes at an arbitrary time *t*_i_, echoes can be interpolated between elevation angles *θ*_j_ and *θ*_j+1_, as shown in Fig. 14(c). An example of elevation interpolation of radar echoes is shown in Fig. 16, where linear interpolation at 0.1° increments of elevation angle was applied to the PPI echoes observed at 16:39 LST from the same eruption used in Fig. 15. The result demonstrated that the interpolated echoes smoothly connect the observed echoes.

### Three-dimensional CAPPI data

**Fig. 17:**
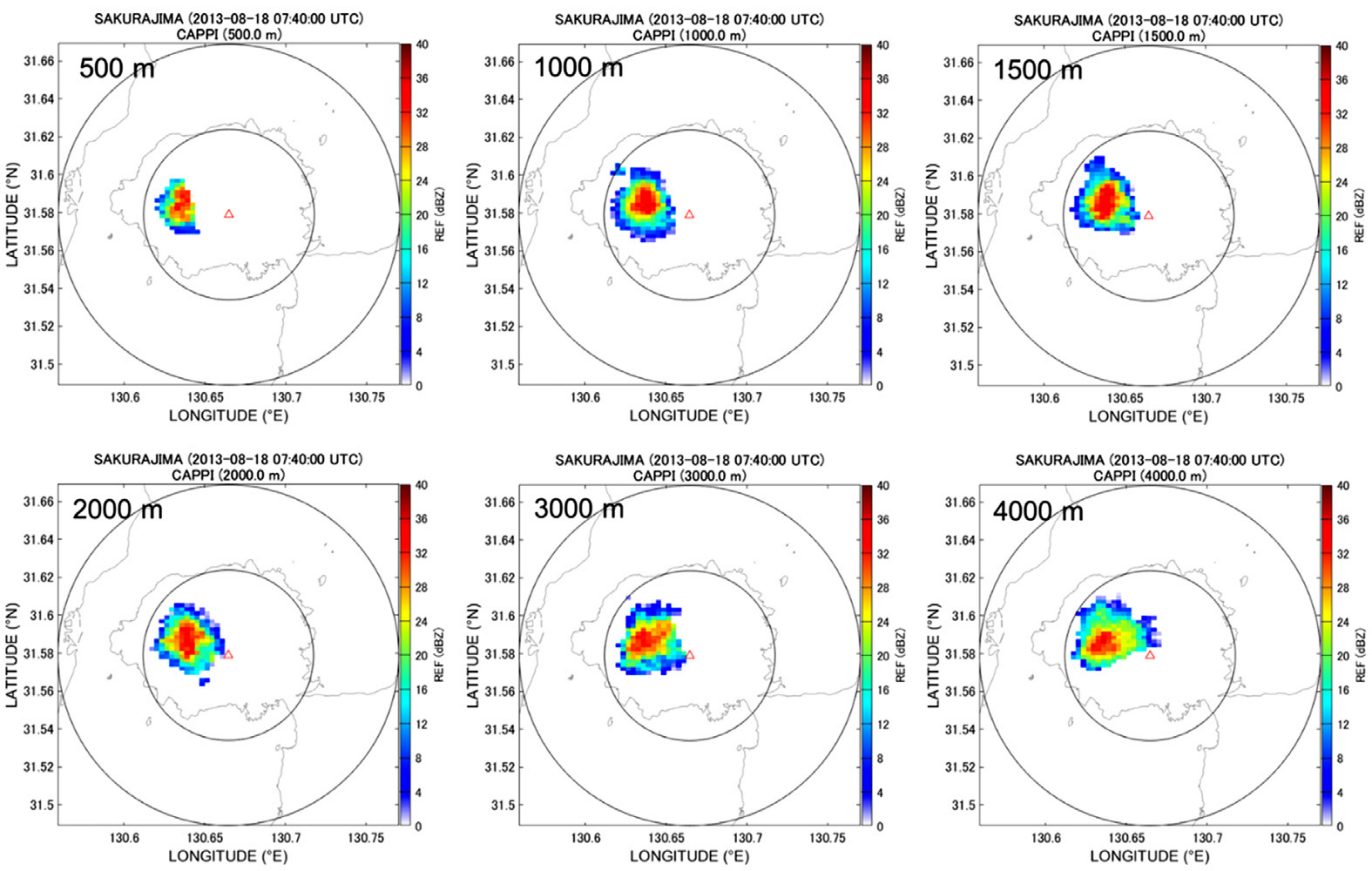
CAPPI images at heights of 500, 1000, 1500, 2000, 3000, and 4000 m, respectively. 16:40 LST, August 18, 2013, Sakurajima eruption.

Radar echoes interpolated in the previous section are expressed in the radar coordinate system. Thus, coordinate system transformation to the geographic coordinate system is necessary to construct three-dimensional CAPPI data and quantitatively examine the volcanic eruption cloud structure. The present study uses Vincenty's direct method [16]. The longitude and latitude resolutions of the geographic coordinate system are set to approximately 100 m; the vertical resolution is also set to 100 m. Fig. 17 shows an example of CAPPI images of *Z* obtained by ANT3D_GUI.

#### Future improvements

ANT3D is still under development through the additions of new functions such as quantitative ash falls estimation (QEA), quantitative ash falls forecasting (QAF), estimation of volcanic ash cloud top height, discrimination of precipitation and ash fall, and mosaic data from multiple radar observations. The present methods were used for a three-dimensional study of the inner structures and top heights of eruption clouds, and the amount and area of ash falls [3]. One of the limitations of the present method may be its application to continuous eruption clouds, because the accuracy of advection vector estimation seems to be worse than other methods of estimation.

## Declaration of Competing Interest

The authors declare that they have no known competing financial interests or personal relationships that could appear to have influenced the work reported in this paper.

## Figures and Tables

**Table A1:** System requirements.

Operating system	Windows 10
Processor	Any Intel or AMD x86-64 processor
Disk	5–8 GB
RAM	8 GB

**Fig. A1:**
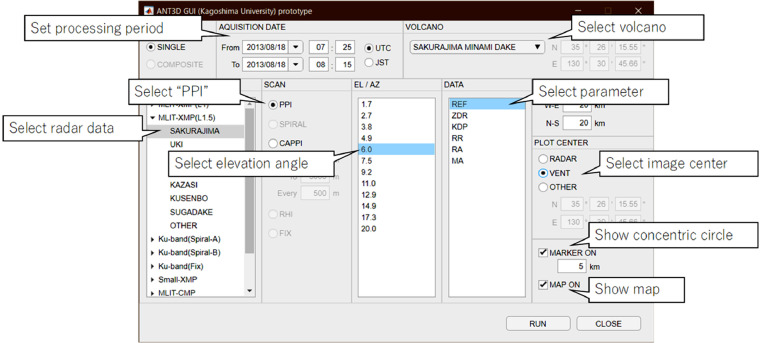
ANT3D_GUI main window (create PPI images).

**Fig. A2:**
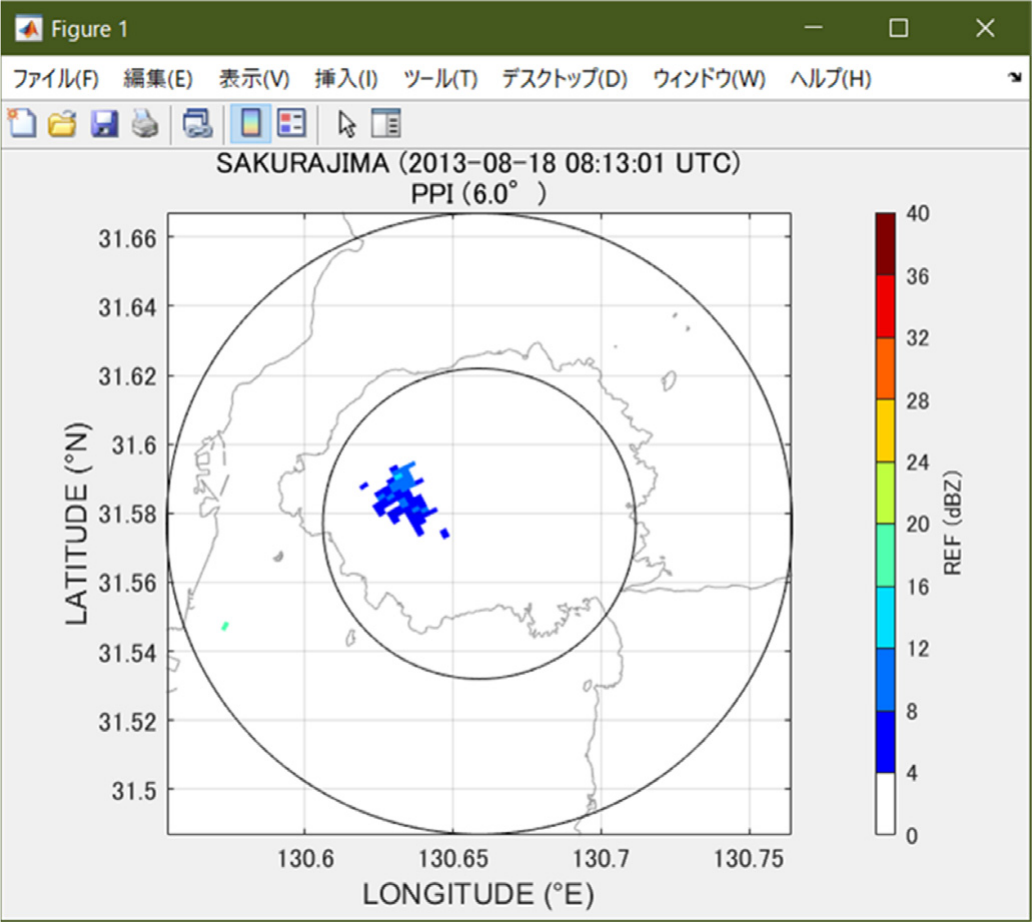
ANT3D_GUI output–PPI image.

**Fig. A3:**
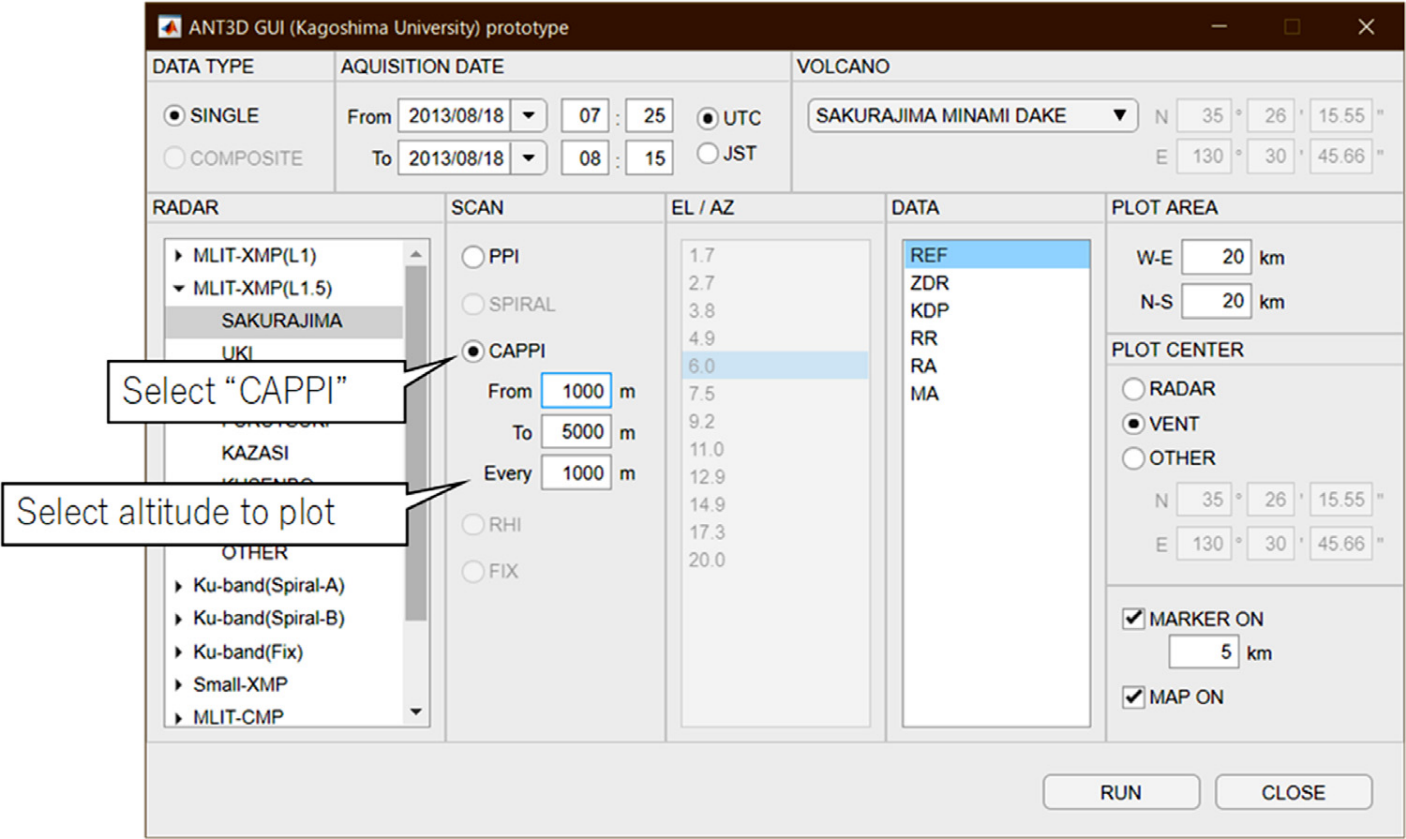
ANT3D_GUI main window (create CAPPI images).

**Fig. A5:**
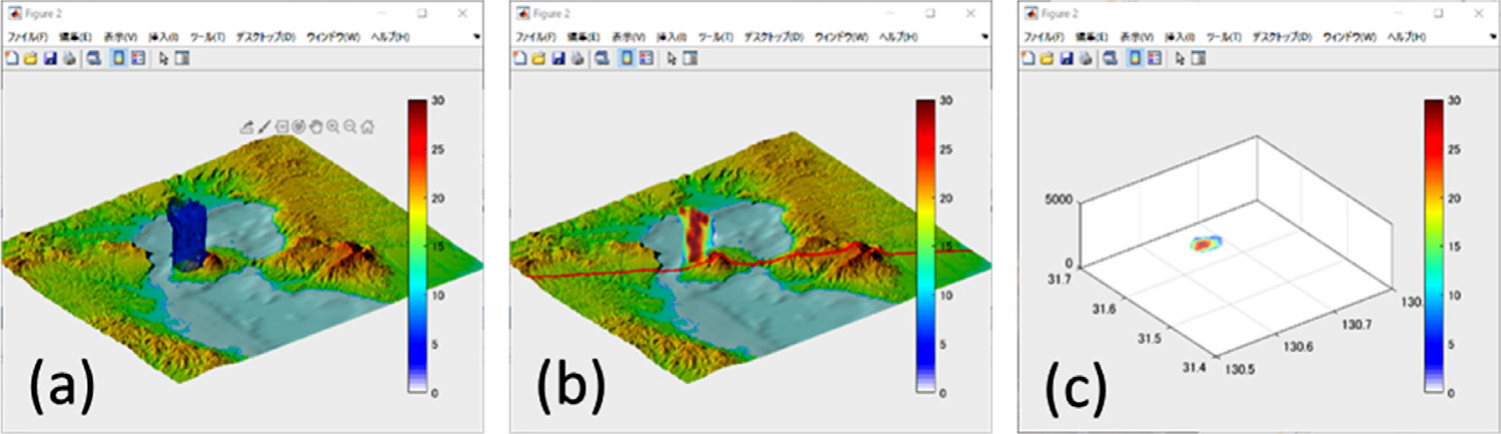
Examples of CAPPI images. (a) isosurface, (b) vertical cross section, and (c) horizontal cross section.

**Fig. A4:**
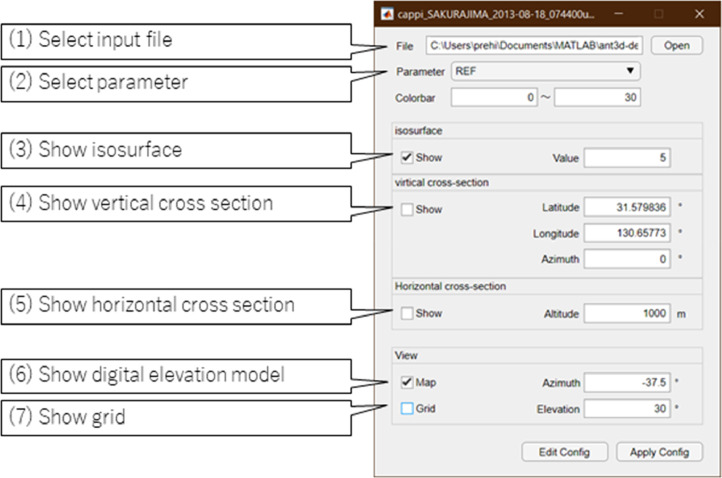
CAPPI viewer main window.

## Appendix A Program ANT3D_GUI and CAPPI viewer

These programs can be downloaded from Mendeley Data [17].

### a. Installation

*System requirements*

Table A1 shows the system requirements for ANT3D_GUI and the CAPPI viewer.

These applications also require MATLAB Runtime. Download the MATLAB Runtime installer (release R2019a for Windows) from the MathWorks website [18] and install it on your computer:

*Installing the applications*

Copy the following directories to your computer.

• ant3d_gui/
• cappiviewer/

b. User's manual

ANT3D_GUI

(1) Preparing input files

The following instructions explain the creation of PPI images and CAPPI images from MLIT X-Band MP Radar Network (XRAIN) data. Copy the input files to the input directory before you launch ANT3D_GUI. The input directory is defined in the ant3d_gui/path.csv file.

(2) Create PPI images

Double-click on ant3d_gui.exe and the main window will appear (Fig. A1). Set the processing condition as in Fig. A1 and click the “Run” button to create PPI images. The images are displayed and saved to the output directory in PNG (.png) format (Fig. A2).

(3) Create CAPPI images

Set the processing condition as in Fig. A3 and click the “Run” button to create CAPPI images. The images are displayed and saved to the output directory in PNG (.png) format. 3D CAPPI data are also saved to the output directory. These 3D CAPPI data can be viewed using the CAPPI viewer (see section A.2.2).

*CAPPI viewer*

Double-click on “cappiviewer.exe” and the main window will appear (see Fig. A4). You can display the isosurface, vertical cross section, and horizontal cross section of the selected parameter by following the instructions below.

(1) Press the “Open” button and select the input file.
(2) Select the parameter from the pull-down menu.
(3) If the “Show” option in the isosurface panel is checked, the isosurface will be shown (Fig. A5(a)). You can specify the value of the isosurface.
(4) If the “Show” option in the vertical cross-section panel is checked, the vertical cross section will be shown (Fig. A5(b)). You can define the vertical cross section by latitude, longitude, and azimuth.
(5) If the “Show” option in the horizontal cross-section panel is checked, the horizontal cross section will be shown (Fig. A5(c)). You can specify the altitude of the horizontal cross section.
(6) If the “Map” option is checked, the digital elevation model will be shown on the Fig..
(7) If the “Grid” option is checked, grid lines will be shown on the Fig..
